# Elevated tRNA halves in olfactory epithelial cells of patients with schizophrenia

**DOI:** 10.1172/JCI195148

**Published:** 2026-01-16

**Authors:** Justin T. Gumas, Megumi Shigematsu, Karin E. Borgmann-Winter, Chang-Gyu Hahn, Yohei Kirino

**Affiliations:** 1 Computational Medicine Center,; 2 Department of Biochemistry and Molecular Biology,; 3 Department of Psychiatry and Human Behavior,; 4 Department of Neuroscience, and; 5 Vickie & Jack Farber Institute for Neuroscience, Sidney Kimmel Medical College, Thomas Jefferson University, Philadelphia, Pennsylvania, USA.

**Keywords:** Cell biology, Public Health, RNA processing, Schizophrenia

## Abstract

Schizophrenia patients’ olfactory epithelial cells contain abundant immune-activating tRNA fragments, linking small RNA biology to inflammation and suggesting avenues for diagnostics.

**To the Editor:** Schizophrenia is a multifactorial neuropsychiatric disorder with limited treatment options (1). Elevated levels of pro-inflammatory cytokines in the blood and cerebrospinal fluid have been implicated in driving inflammation and immune dysregulation within the central nervous system (CNS), which are considered key elements of schizophrenia pathophysiology (1, 2). Microglia, the resident macrophages of the CNS, are a primary source of inflammatory cytokines in the brain and have been proposed as a common cause of neuroinflammation in patients with schizophrenia (1, 2). Endosomal Toll-like receptor 7 (TLR7), which senses short noncoding RNAs (sncRNAs) as its ligands, mediates cytokine induction in microglia and is upregulated in schizophrenia (3). Although TLR7 ligands have traditionally been understood to consist of exogenous RNAs from invading pathogens, our recent studies using human macrophages have identified endogenous sncRNAs, particularly transfer RNA (tRNA) halves, which represent the most abundant species of tRNA-derived sncRNAs, as potent TLR7 activators (4, 5). Among the various tRNA half species generated by the tRNA anticodon endoribonuclease angiogenin (Ang), two specific 5′-tRNA halves derived from tRNA^HisGUG^ and tRNA^ValCAC/AAC^ (5′-half^HisGUG^ and 5′-half^ValCAC/AAC^) have been shown to robustly activate TLR7, inducing the secretion of cytokines, such as IL-1β and TNF-α (4, 5).

In this study, we characterized the expression profiles of tRNA halves, potential contributors to cytokine elevation via the TLR7 axis, in olfactory epithelial (OE) cells derived from individuals with schizophrenia and matched healthy controls. OE cells represent the only neural tissue readily obtainable from living humans via biopsy and thus have been utilized to investigate morphological and molecular alterations in psychiatric disorders, including schizophrenia (6). The olfactory system is susceptible to inflammatory changes, and alterations in olfaction have been linked to schizophrenia. We quantified tRNA halves in OE cells obtained from age- and sex-matched pairs of healthy individuals and patients with schizophrenia. Using our established TaqMan RT-qPCR for specific tRNA half quantification (4), we analyzed all samples in blinded experiments. As shown in Figure 1A, all four examined 5′-tRNA halves were significantly upregulated in OE cells from schizophrenia, including the potent TLR7-stimulating species 5′-half^HisGUG^ and 5′-half^ValCAC/AAC^ (4, 5). Moreover, *Ang* mRNA levels were significantly elevated in the schizophrenia samples (Figure 1B). Since Ang-mediated tRNA cleavage is known to be induced by various factors, including cellular stress, hormone signaling pathways, and immune responses, these findings suggest that pathophysiological factors associated with schizophrenia, present in the olfactory epithelium of the patients, lead to increased Ang expression and enhanced production of immunostimulatory tRNA halves.

To further characterize tRNA halves in the OE cell samples, we performed 2′,3′-cyclic phosphate RNA-Seq (cP-RNA-Seq), which can specifically sequence cP-containing 5′-tRNA halves (4) (Supplemental Figure 1B; supplemental material available online with this article; https://doi.org/10.1172/JCI195148DS1). Profiling of the resulting tRNA-derived reads, based on the tRNA fragment (tRF) classification scheme (Supplemental Figure 1C), revealed no discernable differences in subclass ratios between healthy and schizophrenia samples (Figure 1C). Notably, 5′-derivatives (i.e., 5′-halves, 5′-tRFs, and i-tRFs-5′) dominated over 3′-derivatives, with 5′-halves being the most prevalent. Pearson’s correlation analysis showed strong correlations among the 5′-derivatives and among the 3′-derivatives in both healthy and schizophrenia libraries (Figure 1D). Four tRNA isoacceptors, namely tRNA^GluCUC^, tRNA^LysCUU^, tRNA^ValCAC/AAC^, and tRNA^GlyGCC^, consistently emerged as the primary sources of 5′-derivatives (Figure 1E and Supplemental Figure 1D), with 5′-halves far more abundant than 5′-tRFs and i-tRFs-5′ (Figure 1F). Importantly, the two immunostimulatory 5′-halves were among the major species: 5′-half^ValCAC/AAC^ was one of the top four, and 5′-half^HisGUG^ ranked as the seventh most abundant. The reads from the top four isoacceptors consistently originated from a limited subset of isodecoders (Supplemental Figure 2A). The endonucleolytic cleavage sites for generating 5′-halves and i-tRFs-5′ were largely consistent (Figure 1G and Supplemental Figure 2B). As for 5′-tRFs, while many cleavage positions were located in the double-stranded regions of mature tRNAs (Supplemental Figure 2B), these sites predominantly mapped to the single-stranded regions within the 5′-halves (Supplemental Figure 2C). Collectively, these results support the conclusion that immunostimulatory tRNA halves are abundantly accumulated and that 5′-halves serve as substrates for the cleavages producing shorter 5′-tRFs and i-tRFs-5′.

Although miRNAs have been extensively studied in schizophrenia, growing evidence supports the functional relevance of non-miRNA-sncRNAs, including tRNA halves. Our study adds to this emerging field by identifying significantly elevated immunostimulatory tRNA halves in OE cells from schizophrenia. Two limitations should be noted: the small sample size and antipsychotic treatment in all patients at the time of biopsy. Although OE cells were passaged multiple times, the possibility of long-term effects of medication cannot be fully excluded. Nevertheless, our findings suggest that elevated immunostimulatory tRNA halves may contribute to schizophrenia pathophysiology by inducing cytokines. Given the considerable heterogeneity in schizophrenia diagnosis and clinical presentation, future studies with larger cohorts will be essential to determine whether patient-to-patient variability in tRNA half expression correlates with specific clinical features and whether these alterations persist in patients’ brains. Such investigations will clarify their potential as therapeutic targets or diagnostic biomarkers.

## Funding support

This work is the result of NIH funding, in whole or in part, and is subject to the NIH Public Access Policy. Through acceptance of this federal funding, the NIH has been given a right to make the work publicly available in PubMed Central.

- NIH grants (GM106047, GM156496, HL175371, HL150560, AI168975, and AI171366 to YK).
- NIH grant (MH132097 to KEBW and CH).

## Supplementary Material

Supplemental data

Supporting data values

## Figures and Tables

**Figure 1:**
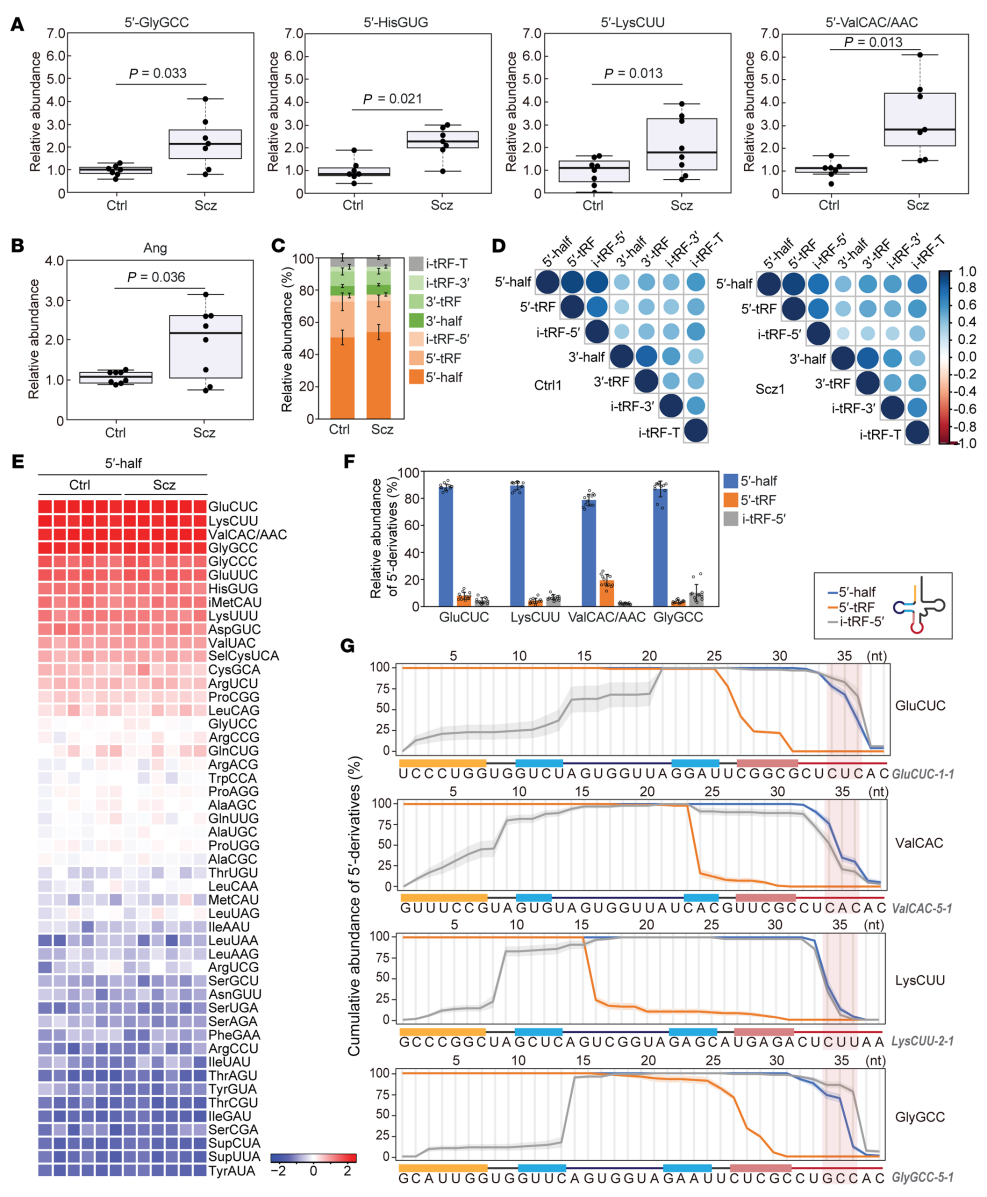
Increased levels of 5′-tRNA halves in OE cells of patients with schizophrenia. (**A** and **B**) Quantification of specific 5′-tRNA halves (**A**) and *Ang* mRNA (**B**) in OE cells. Relative expression levels are shown with one control sample set as 1. After outlier exclusion (see Statistics in Supplemental Methods), sample sizes were 7 for 5′-GlyGCC, 5′-HisGUG, and 5′-ValCAC/AAC, and 8 for 5′-LysCUU and *Ang* mRNA. *P* values were determined using a paired 2-tailed *t* test. (**C**) Average proportions of tRNA-derived sncRNA reads obtained by cP-RNA-Seq. (**D**) Pearson’s correlation analysis among tRNA-derived sncRNA subclasses. The color and size of dots reflect the correlation coefficient, calculated as the *z* score. (**E**) Heatmap of 5′-tRNA half read distributions. Coloration reflects the log_10_ reads per million–based *z* score. (**F**) Average proportions of 5′-derivative reads across all samples. (**G**) Analysis of read positions for each 5′-derivative. The highest read count within each subclass was set to 100%, and the counts at each nucleotide position are shown as relative abundances with SD from all samples shown as shaded regions.

## Version 1 01/16/2026

Electronic publication
