# Supplementary material for: Elevated tRNA halves in olfactory epithelial cells of patients with schizophrenia
Source: J Clin Invest. 2026 Jan 16;136(2):e195148. doi: 10.1172/JCI195148 (PMC12807459; doi:10.1172/JCI195148)
Supplement: Supplemental data [file jci-136-195148-s125.pdf]

## Supplemental Materials

## Methods

### Olfactory epithelial biopsy and human OE cells

Olfactory epithelial biopsies were obtained from individuals with schizophrenia and from age- and sex-matched individuals without psychiatric illness. These subjects were recruited and assessed at the Schizophrenia Research Center of the University of Pennsylvania. Olfactory tissues were obtained in collaboration with the Department of Otorhinolaryngology at the University of Pennsylvania. All subjects were informed of the nature and potential risks of the study and provided written informed consent for participation. All procedures involving human tissue were conducted in accordance with the Declaration of Helsinki and were approved by the Institutional Review Board of the University of Pennsylvania. For olfactory biopsy, the nasal cavity was anesthetized with pontocaine spray, and two 1-mm biopsies were obtained with giraffe forceps: one from the high middle turbinate and the other from the opposed septum. The tissue samples were transferred to culture medium for transport to the laboratory. OE cells were prepared as previously described (1). Upon reaching confluence, the cells were passaged and frozen in cell freezing medium (5% FBS in DMSO), and stored in the vapor phase of a liquid nitrogen freezer at −140°C for subsequent studies. At the time of RNA extraction for this study, the passage number of the cells was three or four. Quantification of mRNA for three neurodifferentiation markers (*TrkB2*, *OcNc1*, and *ACIII*), as described in (1), confirmed their presence in OE cells, with no significant differences between healthy and schizophrenia samples (**Supplementary Fig. S1A**).

**Sex and age as biological variables**

Given that the expression of tRNA halves and other sncRNAs can be modulated by sex hormones (2) and aging (3), we utilized OE cells obtained from age- and sex-matched pairs of healthy individuals and schizophrenia patients (**Supplementary Table S1**).

**TaqMan RT-qPCR for 5′-tRNA half quantification**

Total RNA was isolated from the OE cells using TRIsure (Bioline). The levels of 5′-tRNA halves were quantified using a multiplex TaqMan RT-qPCR method that we developed for specific quantification of 5′-tRNA halves (4). Briefly, 200 ng of total RNA from OE cells was subjected to T4 Polynucleotide Kinase (T4 PNK) treatment and 3′-adapter (AD) ligation as described previously (4). Subsequently, multiplex TaqMan RT-qPCR was performed using One Step PrimeScript RT-PCR Kit (Takara Bio) on a StepOne Plus Real-time PCR machine (Applied Biosystems). The expression levels were normalized to those of U6 snRNA. The sequences of the targeted 5′-tRNA halves, primers, and TaqMan probes have been described previously (4-8). The information on license plates (9) and names via tDRnamer (10) for the targeted 5′-tRNA halves in this study are included in **Supplementary Tables S2**.

**Standard RT-qPCR for mRNA quantification**

For quantification of *Ang* mRNA, total RNA from OE cells was treated with RQ1 DNase (Promega) and subjected to reverse transcription using RevertAid Reverse Transcriptase (Thermo Scientific) and a reverse primer. The synthesized cDNAs were then subjected to PCR using SYBR Green PCR Master Mix (Applied Biosystems) along with forward and reverse

primers on the StepOnePlus Real-Time PCR System (Applied Biosystems). The expression levels were normalized to those of U6 snRNA. The sequences of the primers are shown in (6).

**Statistics**

In **Fig. 1A-B** and **Supplementary Fig. S1A,** after quantifying nine pairs of samples, we excluded outliers defined a priori as values greater than Q3 + 1.5 × IQR or less than Q1 – 1.5 × IQR (where Q1 and Q3 represent the first and third quartiles, and IQR is the interquartile range, Q3 – Q1), and also removed the corresponding matched sample to preserve pairing. After outlier exclusion, the final sample sizes (n) for each panel are reported in the figure legend. *P*-values were determined using a paired two-tailed t-test. For the cP-RNA-seq analysis described below, we analyzed six pairs that passed RT-qPCR QC (no outliers) as a prespecified technical quality criterion.

**cP-RNA-seq and bioinformatics**

Total RNA from OE cells (**Supplementary Table S1**) was subjected to cP-RNA-seq as described previously (2, 3, 6, 8, 11, 12). Briefly, short RNAs were isolated using the mirVana miRNA isolation kit (Thermo Fisher Scientific) and then subjected to sequential treatment with calf intestinal phosphatase, sodium periodate, and T4 PNK. The treated RNA was subsequently used for cDNA amplification with the TruSeq Small RNA kit (Illumina). The amplified cDNAs were gel-purified, and their quality and quantity were assessed using a Bioanalyzer High Sensitivity DNA chip (Agilent) and Qubit (Thermo Fisher Scientific). The cDNA libraries were sequenced on Illumina NovaSeq 6000 platform by Genewiz (Azenta Life Sciences). Bioinformatic analyses were performed as described previously (3, 5, 7, 8). In brief, we utilized

the cutadapt tool (DOI: http://dx.doi.org/10.14806/ej.17.1.200) to remove the 3′-AD. After selecting 15–60-nt reads, we used Bowtie2 (2.3.5) (13) for mapping. Data analysis and visualization were carried out using *R* packages: *gplots* for heat map analysis and *corrplot* for *Pearson* correlation analysis. Importantly, in this study, cP-RNA-seq was applied to profile the repertoire and within-sample composition of tRNA-derived sncRNAs, rather than to estimate relative abundance across samples, as our analysis did not utilize absolute normalization references.

**Study approval**

All procedures involving human tissue were conducted in accordance with the Declaration of Helsinki and approved by the Institutional Review Board of the University of Pennsylvania. The Office of Human Research (OHR) of Thomas Jefferson University (TJU) also approved our use of human samples without any private information, adhering to all federal, institutional, and ethical guidelines.

**Data availability**

The obtained sequence reads are publicly available from the NCBI Sequence Read Archive (BioProject: PRJNA1238227).

**Acknowledgments**

We are grateful to the members of the Kirino lab for helpful discussions. This study was supported in part by National Institutes of Health Grant (GM106047, GM156496, HL175371, HL150560, AI168975, and AI171366 to Y.K., and MH132097 to K.E.B-W. and C.H.).

## Author contributions

The project was conceived by Y.K. in consultation with K.E.B-W. and C.H. K.E.B-W. and C.H. cultured OE cells and provided total RNA from these cells to J.G. and Y.K. J.G. performed the remaining experiments and conducted sequencing and bioinformatics analyses with guidance from M.S. The manuscript was written by J.G. and Y.K. with contributions from K.E.B-W., C.H., and M.S. Funding support was provided by K.E.B-W., C.H., and Y.K.

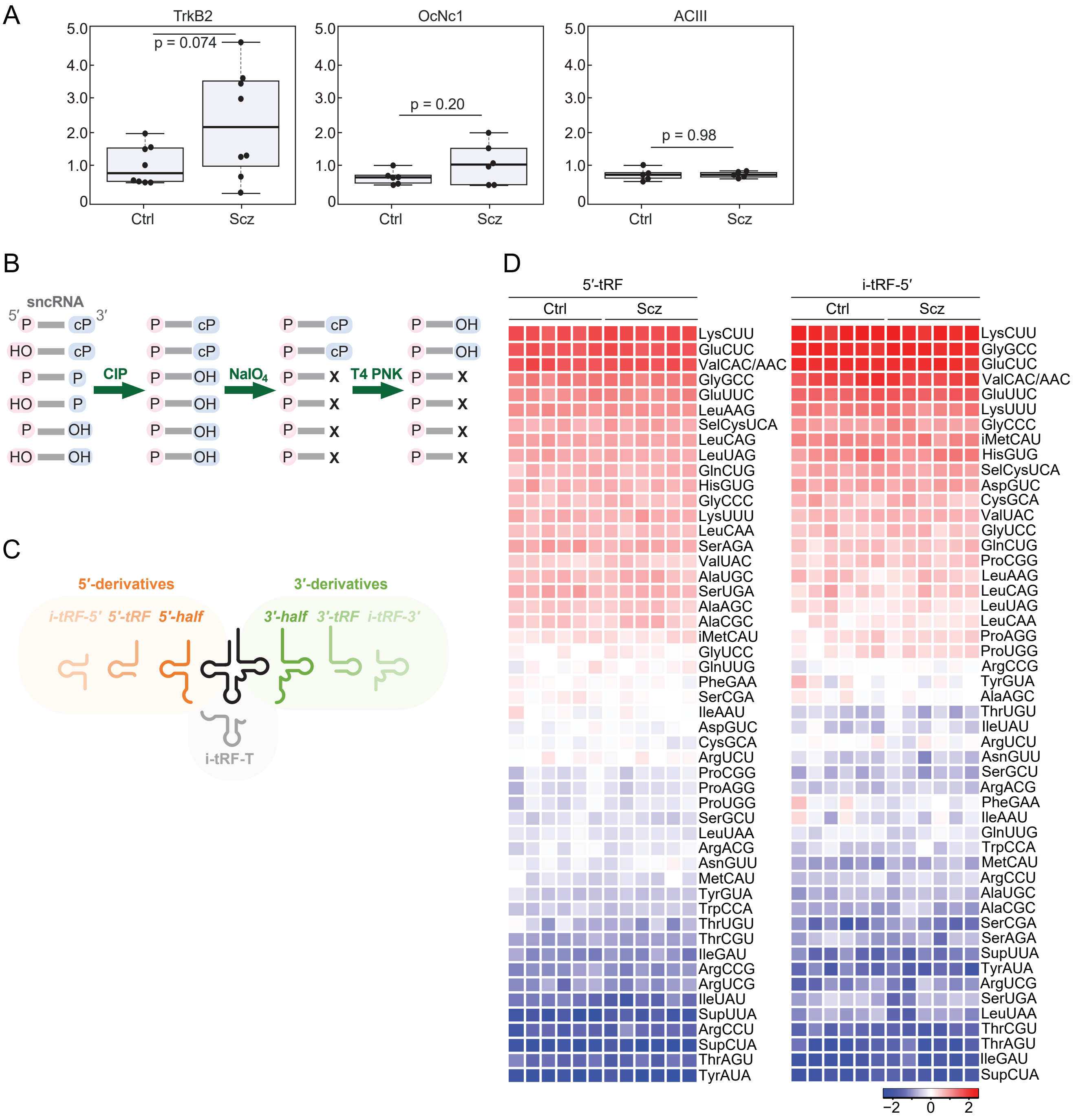


**Figure S1. Characterization of tRNA-derived reads obtained by cP-RNA-seq**

**(A)** Quantification of the indicated three mRNAs in OE cells. Relative expression levels are shown with one control sample set as 1. After outlier exclusion (see Statistics in the Supplementary Methods), sample sizes were eight for *TrkB2*, six for *OcNc1*, and five for *ACIII*. *P*-values were determined by means of two-tailed Student's t-test. **(B)** Schematic representation of the cP-RNA-seq procedure. **(C)** Schematic representation of the different classes of tRNA-derived sncRNAs, categorized using the tRF classification method (14-16) with modifications incorporated from our previous studies (3, 5, 7). tRNA halves are produced by anticodon-loop cleavage, with the 5′- and 3′-halves retaining their respective intact ends. tRFs encompass all other tRNA-derived sncRNAs; 5′-tRFs and 3′-tRFs maintain the mature 5′- and 3′-ends, respectively, while internal tRFs (i-tRFs) are derived solely from the internal regions of mature tRNAs. We further subclassified i-tRFs into i-tRFs-5′, i-tRFs-3′, and i-tRFs-T, originating from the 5′-region, the 3′-region, and the region encompassing the entire anticodon-loop, respectively. **(D)** Heatmap showing the read distribution for 5′-tRF and i-tRF-5′ from the respective cyto tRNA isoacceptors. Coloration reflects the $\log_{10}$ RPM-based z-score.

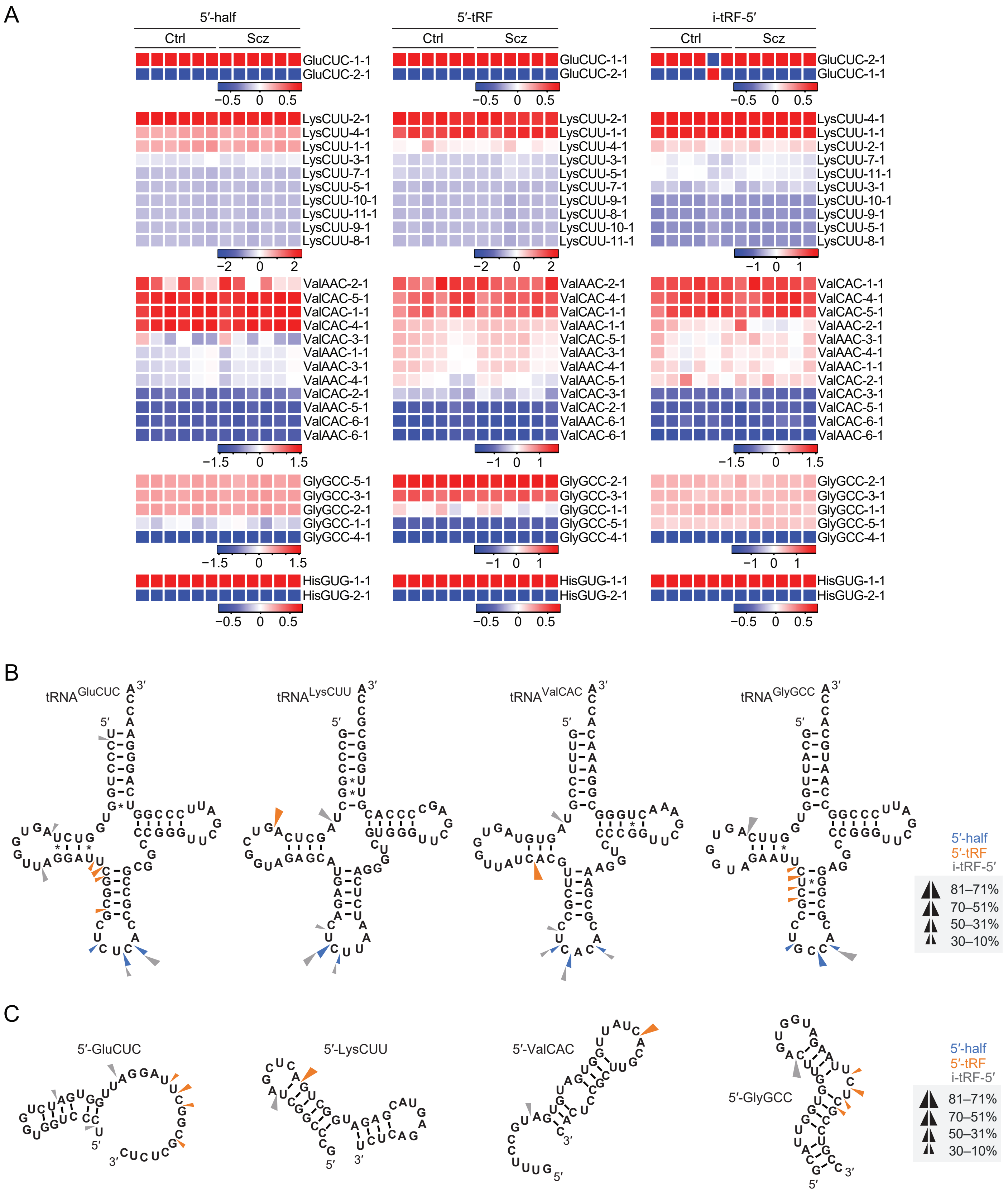


**Figure S2. Characterization of cleavage sites in the generation of tRNA-derived sncRNAs**

**(A)** Heatmap showing the read distribution for 5′-derivatives derived from the respective isodecoders. Coloration reflects the RPM-based z-score. **(B, C)** Cleavage sites in the indicated mature tRNAs (A) and 5′-halves (B), predicted based on the terminal positions of the identified 5′-halves (blue arrowheads), 5′-tRFs (orange arrowheads), and i-tRFs-5′ (gray arrowheads).

**Table S1. Donor information for OE cells used in the cP-RNA-seq study**

| ID | Condition | Sex | Age | Race | Medication |
|---|---|---|---|---|---|
| Ctrl1 | Healthy | Male | 48 | African American | None |
| Ctrl2 | Healthy | Male | 48 | African American | None |
| Ctrl3 | Healthy | Female | 47 | Asian | None |
| Ctrl4 | Healthy | Female | 52 | Asian | None |
| Ctrl5 | Healthy | Male | 57 | Asian | None |
| Ctrl6 | Healthy | Female | 30 | Caucasian | None |
| Scz1 | Schizophrenia | Male | 46 | African American | Risperidone, Prolixin |
| Scz2 | Schizophrenia | Male | 49 | Caucasian | Unknown |
| Scz3 | Schizophrenia | Female | 47 | African American | Haloperidol |
| Scz4 | Schizophrenia | Female | 46 | African American | Haloperidol |
| Scz5 | Schizophrenia | Male | 58 | Caucasian | Unknown |
| Scz6 | Schizophrenia | Female | 31 | African American | Olanzapine |

**Table S2. Unique ID for the 5'-tRNA halves investigated in this study**

| Name | Sequence (5′-to-3′) | License plate | tDRname |
|---|---|---|---|
| 5'-GluCUC | UCCCUGGUGGUCUAGUGGUUAGGAUUCGGCGCUC | tRF-34-87R8WP9N1EWJI5 | tDR-1:34-Glu-CTC-1-M2 |
| 5'-GlyGCC | GCAUUGGUGGUUCAGUGGUAGAAUUCUCGCCUGC | tRF-34-PNR8YP9LON4VHM | tDR-1:35-Gly-GCC-2-M3 |
| 5'-HisGUG | GCCGUGAUCGUAUAGUGGUUAGUACUCUGCGUUG | tRF-34-PW5SVP9N15WV2P | tDR-1:34-His-GTG-1 |
| 5'-LysCUU | GCCCGGCUAGCUCAGUCGGUAGAGCAUGGGACUC | tRF-34-PSQP4PW3FJIKE5 | tDR-1:34-Lys-CTT-1-M2 |
| 5'-ValCAC/AAC | GUUUCCGUAGUGUAGUGGUUAUCACGUUCGCCU | tRF-33-79MP9P9NH57SD3 | tDR-1:33-Val-AAC-1-M6 |

## References for Supplemental Materials


1. Borgmann-Winter KE, Rawson NE, Wang HY, Wang H, Macdonald ML, Ozdener MH, et al. Human olfactory epithelial cells generated in vitro express diverse neuronal characteristics. *Neuroscience.* 2009;158(2):642-53.
2. Honda S, Loher P, Shigematsu M, Palazzo JP, Suzuki R, Imoto I, et al. Sex hormone-dependent tRNA halves enhance cell proliferation in breast and prostate cancers. *Proc Natl Acad Sci U S A.* 2015;112(29):E3816-25.
3. Shigematsu M, Morichika K, Kawamura T, Honda S, and Kirino Y. Genome-wide identification of short 2',3'-cyclic phosphate-containing RNAs and their regulation in aging. *PLoS Genet.* 2019;15(11):e1008469.
4. Kawamura T, Shigematsu M, and Kirino Y. In vitro production and multiplex quantification of 2',3'-cyclic phosphate-containing 5'-tRNA half molecules. *Methods.* 2021.
5. Gumas J, Kawamura T, Shigematsu M, and Kirino Y. Immunostimulatory short non-coding RNAs in the circulation of patients with tuberculosis infection. *Mol Ther Nucleic Acids.* 2024;35(1):102156.
6. Pawar K, Shigematsu M, Sharbati S, and Kirino Y. Infection-induced 5'-half molecules of tRNAHisGUG activate Toll-like receptor 7. *PLoS Biol.* 2020;18(12):e3000982.
7. Shigematsu M, Kawamura T, Deshpande DA, and Kirino Y. Immunoactive signatures of circulating tRNA- and rRNA-derived RNAs in chronic obstructive pulmonary disease. *Mol Ther Nucleic Acids.* 2024;35(3):102285.
8. Shigematsu M, and Kirino Y. Oxidative stress enhances the expression of 2',3'-cyclic phosphate-containing RNAs. *RNA Biol.* 2020;17(8):1060-9.
9. Pliatsika V, Loher P, Telonis AG, and Rigoutsos I. MINTbase: a framework for the interactive exploration of mitochondrial and nuclear tRNA fragments. *Bioinformatics.* 2016;32(16):2481-9.
10. Holmes AD, Chan PP, Chen Q, Ivanov P, Drouard L, Polacek N, et al. A standardized ontology for naming tRNA-derived RNAs based on molecular origin. *Nat Methods.* 2023;20(5):627-8.
11. Honda S, Morichika K, and Kirino Y. Selective amplification and sequencing of cyclic phosphate-containing RNAs by the cP-RNA-seq method. *Nat Protoc.* 2016;11(3):476-89.
12. Shigematsu M, Kawamura T, Morichika K, Izumi N, Kiuchi T, Honda S, et al. RNase kappa promotes robust piRNA production by generating 2',3'-cyclic phosphate-containing precursors. *Nat Commun.* 2021;12(1):4498.
13. Langmead B, and Salzberg SL. Fast gapped-read alignment with Bowtie 2. *Nat Methods.* 2012;9(4):357-9.
14. Lee YS, Shibata Y, Malhotra A, and Dutta A. A novel class of small RNAs: tRNA-derived RNA fragments (tRFs). *Genes Dev.* 2009;23(22):2639-49.
15. Wilson B, and Dutta A. Function and Therapeutic Implications of tRNA Derived Small RNAs. *Front Mol Biosci.* 2022;9:888424.
16. Magee R, and Rigoutsos I. On the expanding roles of tRNA fragments in modulating cell behavior. *Nucleic Acids Res.* 2020;48(17):9433-48.
